# Connexin 43 Channels in Osteocytes Regulate Bone Responses to Mechanical Unloading

**DOI:** 10.3389/fphys.2020.00299

**Published:** 2020-03-31

**Authors:** Dezhi Zhao, Ruofei Liu, Guobin Li, Meng Chen, Peng Shang, Hui Yang, Jean X. Jiang, Huiyun Xu

**Affiliations:** ^1^Key Laboratory for Space Bioscience and Biotechnology, School of Life Sciences, Northwestern Polytechnical University, Xi’an, China; ^2^Key Laboratory for Space Bioscience and Biotechnology, Research and Development Institute in Shenzhen, Northwestern Polytechnical University, Shenzhen, China; ^3^Research Center of Special Environmental Biomechanics and Medical Engineering, Northwestern Polytechnical University, Xi’an, China; ^4^Department of Biochemistry and Structural Biology, The University of Texas Health Science Center, San Antonio, TX, United States

**Keywords:** Cx43, gap junction, hemichannel, hindlimb unloading, osteocyte

## Abstract

Connexin (Cx) 43 forms gap junctions and hemichannels that mediate communication between osteocytes and adjacent cells or the extracellular environment in bone, respectively. To investigate the role of each channel type in response to mechanical unloading, two transgenic mouse models overexpressing dominant-negative Cx43 predominantly in osteocytes driven by a 10 kb dentin matrix protein 1 (*Dmp1*) promoter were generated. The R76W mutation resulted in gap junction inhibition and enhancement of hemichannels, whereas the Δ130–136 mutation inhibited both gap junctions and hemichannels. Both mutations led to cortical bone loss with increased endocortical osteoclast activity during unloading. Increased periosteal osteoclasts with decreased apoptotic osteocytes were observed only in R76W mice. These findings indicated that inhibiting osteocytic Cx43 channels promotes bone loss induced by unloading, mainly in the cortical area; moreover, hemichannels protect osteocytes against apoptosis and promote periosteal bone remodeling, whereas gap junctions modulate endocortical osteoclast activity in response to unloading.

## Introduction

Bone structure adapts to the mechanical environment. Mechanical unloading caused by microgravity during long space flight leads to bone mineral density (BMD) loss at a rate of 0.5–1.5% per month that is only partly restored 1 year after return to Earth (Lang et al., 2006). This phenomenon is caused by the absence of normal mechanical stimulation at zero gravity, which results in inadequate anabolic signaling for bone formation and increased bone resorption (Lin et al., 2009; Jing et al., 2014). However, the cellular mechanisms underlying the bone loss induced by unloading remain unclear.

Osteocytes constitute the major mechanosensory cells in bone tissue (Pavalko et al., 2003). Long dendritic processes connect neighboring osteocytes and other bone cells to form a network (Bonewald, 2005), which responds to and transfers mechanical signals and regulates the behavior of osteoblasts and osteoclasts (Jacobs et al., 2010). Hemichannels in addition to gap junctions formed by connexins (Cxs) play important roles in this process by allowing the passage of small molecules (≤1 kDa) between adjacent cells and between cells and the extracellular environment. As the most abundant Cx in osteocytes (Civitelli, 2008), Cx 43 is highly responsive to mechanical stimulation *in vitro* (Cheng et al., 2001; Thi et al., 2003); accordingly, Cx43-deficient bone cells are less responsive to biomechanical signals (Saunders et al., 2001). *In vivo*, various animal models have been developed to investigate the function of Cx43 in the response to mechanical load. However, the different Cx43 loss-of-function models have yielded variable results (Tatsumi et al., 2007; Grimston et al., 2008, 2011; Zhang et al., 2011; Lloyd et al., 2012, 2013; Bivi et al., 2013). For example, collagen α1 [Colα1(I)]-Cre;Cx43^–/flox^ mice exhibit attenuated anabolic response to mechanical loading on the tibial endocortical surface, as indicated by decreased endocortical bone formation (Grimston et al., 2006, 2008). However, deletion of Cx43 in OC-Cre;Cx43^flox/flox^ (Zhang et al., 2011), DM1-Cre; Cx43^–/flox^ (Grimston et al., 2012), and DMP1-Cre;Cx43^flox/flox^ (Bivi et al., 2013) mice display enhanced anabolic response to mechanical loading, indicated by a greater increase in periosteal bone formation.

Moreover, OC-Cre;Cx43^flox/flox^ mice exhibit reduced response to hindlimb suspension unloading in trabecular but not in cortical areas (Lloyd et al., 2012). This is inconsistent with the study in which Colα1(I)-Cre;Cx43^–/flox^ mice lacking Cx43 demonstrated less sensitivity in cortical bone albeit similar response in trabecular bone as that of wild-type (WT) mice in response to unloading induced by botulinum toxin A injection into hindlimb muscle (Grimston et al., 2011). Together, these findings suggest that Cx43 is involved in the adaptive response to bone loading and unloading; however, the discrepancies in the results highlight the complexity of Cx43 function in bone.

Cx43 forms both gap junction channels and hemichannels at the cell surface. Transgenic mouse models previously generated in our laboratory that overexpress dominant negative Cx43 mainly in osteocytes under the control of the 10 kb dentin matrix protein 1 (*Dmp1*) promoter revealed that R76W mice display blocked gap junctions albeit enhanced hemichannels whereas in Δ130–136 mice, both gap junctions and hemichannels are inhibited (Xu et al., 2015). Compared to WT and R76W mice, 4-month-old Δ130–136 animals present higher femur BMD, expanded marrow cavity, increased osteocyte apoptosis, and poorer material properties but show no obvious alterations in trabecular bone, whereas R76W mice exhibit increased serum levels of bone remodeling markers (Xu et al., 2015).

In the present study, we utilized these two transgenic models in combination with a well-established hindlimb unloading (HLU) model to investigate contribution of each channel type to the effects of mechanical unloading (Jia et al., 2014). As the functional contribution of osteocytic Cx43 gap junctions and hemichannels among genotypes have been reported previously (Xu et al., 2015), herein we focused on the changes in bone parameters elicited by unloading within each genotype.

## Materials and Methods

### Transgenic Mice

Previously generated Cx43 R76W and Δ130–136 transgenic mouse lines were used in this study (Xu et al., 2015). The transgenes were driven by a 10 kb *Dmp1* promoter in the pBluScript plasmid, resulting in mutant *Cx43* gene expression predominantly in osteocytes. The 3′ end of *Cx43* cDNA linked to a green fluorescent protein cDNA was cloned downstream of an intronic sequence (Xu et al., 2015). The WT and transgenic mice were on a C57BL/6J background. The mice were housed in a temperature-controlled room at 25°C and 40% humidity on a 12:12 h light/dark cycle in the Animal Research Lab of Northwestern Polytechnical University under specific pathogen-free conditions. Food and water were freely available. Male 10 week old transgenic and WT mice were used for experiments. Genotyping was performed by real-time polymerase chain reaction (PCR) using genomic DNA isolated from mouse toes (Xu et al., 2015). All animal protocols were approved by the Northwestern Polytechnical University Institutional Animal Care and Use Committee.

### Hindlimb Unloading (HLU)

Hindlimb unloading was carried out as previously described (Morey-Holton and Globus, 2002) with some modifications. Briefly, the mouse tail was fixed to a U-shaped copper wire with medical tape. A small plastic pipe was used to cover the mouse tail to protect it from chewing. Littermates were randomly divided into HLU and control groups (*n* = 6–10 per group) and were singly housed. One mouse per cage was subjected to HLU at an angle of 30° to the ground, which maintains normal forelimb load with minimal tail tension and allows the mouse free access to food and water (Jia et al., 2014). Age and genotype-matched non-HLU controls were allowed normal cage activity under identical conditions. Mice were intraperitoneally injected with 10 mg/kg calcein (Sigma–Aldrich, St. Louis, MO, United States) 14 or 3 days prior to euthanasia on day 28. After 4 weeks of HLU treatment, mice were under isoflurane anesthesia and the tibia and femur were removed for analysis.

### Micro-Computed Tomography (μCT) Analysis

The left femur was isolated and stored in 80% alcohol at 4°C. The femur was fixed in a 20 mm diameter sample tube and the microstructure was examined by high-resolution micro-computed tomography (μCT; GE Healthcare, Madison, WI, United States) at an 8 μm scan resolution with the following settings: 80 kV, 80 μA, 180° total rotation angle, 0.4° rotation step, 2960 ms exposure time, four-frame averaging, and 1 × 1 pixel matrix. After scanning, three-dimensional images were generated using MicroView v.2.1.2 software (GE Healthcare, Madison, WI, United States). The volume of interest of the trabecular metaphysis started 400 μm from the end of the growth plate and extended 1 mm distally, which avoided the primary spongiosa. Cortical parameters were quantified based on a 0.5 mm region of the femoral midshaft (at 55% of length from proximal to distal). Data were analyzed using the MicroView program. Trabecular parameters included bone volume to trabecular volume (BV/TV), trabecular thickness (Tb.Th), trabecular separation (Tb.Sp), trabecular number (Tb.N), and structure model index (SMI). Cortical parameters comprised bone area (B.Ar), BMD, cortical thickness (Ct.Th), marrow area (M.Ar), endocortical surface perimeter (Ec.Pr), and total cross-sectional area inside the periosteal envelope (T.Ar).

### Histological Analysis

The right tibia (*n* = 5–8/group) with soft tissue was isolated and fixed in 4% paraformaldehyde for two days, decalcified in 10% ethylenediaminetetraacetic acid, and embedded in paraffin. Longitudinal sections of the whole bone (5 μm thickness) were made as close to the center of the bone as possible, and at least three tissue sections per tibia were prepared for hematoxylin and eosin staining, tartrate-resistant acid phosphatase staining, and immunohistochemistry analysis as described below. Images were captured using an optical microscope (Model 80i; Nikon, Tokyo, Japan). The number of osteocytes (N.Ot), number of osteoblasts (N.Ob), number of empty lacunae, osteoclast surface (Oc.S), osteoclast number (N.Oc), B.Ar, and bone surface (BS) were quantified using ImageJ software (National Institutes of Health, Bethesda, MD, United States). N.Ot/B.Ar, Oc.S/BS, N.Oc/BS, and N.Ob/BS ratios were calculated. The left femur was isolated and fixed in 80% alcohol at 4°C for 2 days and embedded in methylmethacrylate. At least three sections per tibia were cut longitudinally at a thickness of 50 μm as close to the center of the bone as possible and imaged using a fluorescence microscope (Model 80i). Cortical BS, single label perimeter, double label perimeter, and width between the two labels were quantified using ImageJ software. The mineral apposition rate (MAR), mineralizing surface per BS (MS/BS), and bone formation rate (BFR) were calculated.

### Immunohistochemistry and Terminal Deoxynucleotidyl Transferase dUTP Nick End Labeling (TUNEL) Staining

Paraffin sections of the right tibia (*n* = 3/group) were deparaffinized in toluene and alcohol and then incubated in citric acid antigen retrieval buffer (Servicebio, Wuhan, China) for 8 min in a microwave. The longitudinal sections as close to the center of the bone as possible were incubated in 3% hydrogen peroxide solution for 25 min at room temperature (22°C) to quench intrinsic peroxidase activity. To prevent non-specific binding of antibodies, sections were incubated in Serum-Free Protein Block solution (Solarbio, Beijing, China; A8020) for 30 min prior to immunostaining overnight at 4°C with antibodies against sclerostin (Sigma–Aldrich; SA1126CA), transforming growth factor β1 (TGF-β1) (Sigma–Aldrich; SAB4502954, 1:300), receptor activator of nuclear factor-κB ligand (RANKL) (Servicebio; G1202, 1:100), osteoprotegerin (OPG) (Santa Cruz Biotechnology, Dallas, TX, United States; L112, 1:100), or cleaved caspase-3 (Servicebio; G23303, 1:200). Primary antibodies were detected with anti-rabbit IgG (Proteintech Group, Wuhan, Hubei, China) for 50 min followed by visualization using a diaminobenzidine-horseradish peroxidase substrate detection system (G1211, Servicebio). Nuclei were counterstained with hematoxylin. The ratio of RANKL-positive/OPG-positive osteocytes was combined from RANKL-positive and OPG-positive osteocytes. The *In Situ* Cell Death Detection Kit (Roche, Pleasanton, CA, United States; #11684817910) was used for detection and quantification of cell apoptosis following the manufacturer’s instructions. Briefly, paraformaldehyde-fixed sections were treated with proteinase K (Servicebio; #G1205) for 25 min at room temperature and then incubated in film breaking agent (Servicebio; #G1204) for 20 min at room temperature. Sheared DNAs were labeled with transferase dUTP nick end labeling (TUNEL) reaction mixture with 4′,6-diamidino-2-phenylindole for counterstaining the nucleus (Servicebio; #G1012). The sections were analyzed and photographed using a fluorescence microscope (Model 80i), and the number of apoptotic cells was counted.

### Evaluation of Bone Biomechanical Properties

The right femur was enclosed with a piece of gauze that was pre-soaked in physiological saline at −80°C until analysis. The three-point bending test was performed on a mechanical device (MTS Systems, Eden Prairie, MN, United States; MT5304-30KN). The femur was fixed on two support points 10 mm apart, with the physiological curvature facing upward so that the loading occurred in the mediolateral direction (Leppänen et al., 2006). The loading plate was positioned perpendicular to the long axis of the sample and displacement was applied at a constant rate of 0.5 mm/s. Parameters of the fracture surface were measured using a three-dimensional super-resolution microscope (Hirox-USA, Hackensack, NJ, United States; KH-8700). Elastic modulus, maximum load, and ultimate load were calculated from the stress–strain curve and fracture surface parameters (Jepsen et al., 2015).

### Isolation of RNA and Real-Time PCR

The long-bone tissues of left tibias were isolated free of soft tissue and stored in liquid nitrogen. Bone marrow was removed by flushing with RNase-free water, stored in TRIzol (Ambion, Carlsbad, CA, United States), and pulverized using a tissue homogenizer (Kinematica Polytron, PT 2100). After 15 min dissociation at room temperature, total RNA was extracted and transcribed using HiScript II Q RT supermix for qPCR (Vazyme, Nanjing, JiangSu, China; R223-01). Real-time PCR analysis was performed using the SYBR green PCR kit according to the manufacturer’s instruction (Vazyme; Q311-01). *Gapdh* was used as a housekeeping gene control and expression was calculated by ΔΔCt method using the WT-control group as second reference (Livak and Schmittgen, 2001). The forward and reverse primers used were: 5′-CAA ACT TTT TCA GAG GGG ATC-3′ and 5′-GCA TAC TGT TTC AGC ATG GCA-3′ for caspase-3, and 5′-CGT GCC GCC TGG AGA AAC C-3′ and 5′-TGG AAG AGT GGG AGT TGC TGT TG-3′ for *Gapdh*.

### Statistical Analysis

The homogeneity of variance was analyzed with the Levene test using SPSS v.13.0 software (SPSS Inc., Chicago, IL, United States). And statistical analysis was performed using GraphPad Prism7 statistics software (La Jolla, CA, United States). All data are presented as the means ± SD. Two-way ANOVA with Bonferroni test was used to assess difference between control and suspended within a genotype, and one-way ANOVA with Turkey test was used for multiple group comparisons. Asterisks indicate the degree of significant differences (^∗^*P* < 0.05; ^∗∗^*P* < 0.01; ^∗∗∗^*P* < 0.001).

## Results

### Impaired Cx43 Channels Affect the Response of Cortical Bone to Mechanical Unloading

**FIGURE 1:**
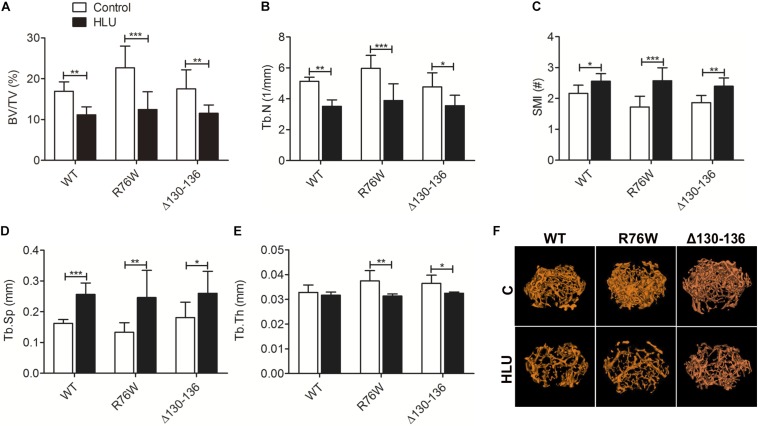
Femoral trabecular bone loss caused by 4 weeks of HLU in WT and Cx43 transgenic mice. μCT analysis reveals decreases in BV/TV **(A)** and Tb.N **(B)** and increases in SMI **(C)** and Tb.Sp **(D)**; Tb.Th **(E)** is decreased in mutant but not in WT mice. Comparisons via two-way with Bonferroni test **(A–E)**. Data represent the means ± SD. *n* = 6–10/group. *P < 0.05, **P < 0.01, ***P < 0.001. **(F)** μCT images of femoral trabecular microstructure. HLU, hindlimb unloading; WT, wild-type; μCT, micro-computed tomography; R, R76W; Δ, Δ130–136; BV/TV, bone volume fraction; Tb.N, trabecular number; SMI, structure model index; Tb.Sp, trabecular separation; Tb.Th, trabecular thickness.

Hindlimb unloading for 4 weeks resulted in a significant loss of femoral distal metaphysis trabecular bone in all mice, which confirmed that the unloading model was established (Figure 1F). μCT analysis revealed a decrease in BV/TV (Figure 1A) and Tb.N (Figure 1B) and increase in SMI (Figure 1C) and Tb.Sp (Figure 1D). Notably, Tb.Th was decreased in the mutants but not in WT mice (Figure 1E). These results suggested that impairment of gap junctions and hemichannels does not significantly affect the mechanical response of trabecular bone to HLU except for Tb.Th.

**FIGURE 2:**
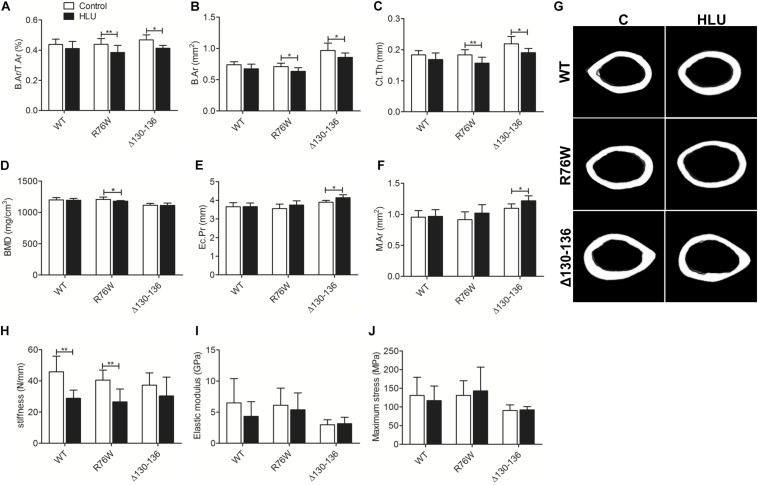
Femoral cortical bone loss in Cx43 transgenic but not in WT mice following HLU. B.Ar/T.Ar **(A)**, B.Ar **(B)**, and Ct.Th **(C)** are decreased in R76W and Δ130–136 transgenic mice; BMD **(D)** is decreased only in R76W mice; and Ec.Pr **(E)** and M.Ar **(F)** are increased only in Δ130–136 mice; **(G)** μCT images of femoral cortical microstructure. Three-point bending assay showing stiffness **(H)** is decreased in WT and R76W mice; elastic modulus **(I)** and maximum stress **(J)** did not differ significantly between control and HLU. Comparisons via two-way with Bonferroni test **(A–F,H–J)**. Data represent the means ± SD. *n* = 6–10/group. *P < 0.05, **P < 0.01. HLU, hindlimb unloading; WT, wild-type; R, R76W; Δ, Δ130–136; B.Ar, bone area; T.Ar, total area; Ct.Th, cortical thickness; BMD, bone mineral density; Ec.Pr, inner perimeter; M.Ar, marrow area; μCT, micro-computed tomography.

In contrast, HLU did not cause significant bone loss in the midshaft cortical bone of WT mice (Figure 2G); however, B.Ar/T.Ar (Figure 2A), B.Ar (Figure 2B), and Ct.Th (Figure 2C) were reduced in both mutants. BMD (Figure 2D) was decreased in R76W but not Δ130–136 mice, whereas Ec.Pr (Figure 2E) and M.Ar (Figure 2F) were increased in Δ130–136 but not R76W mice.

Mechanical testing by three-point bending analysis of the femur revealed significantly decreased stiffness in WT (−37.2%) and R76W mice (−33.2%) during HLU (Figure 2H). However, HLU did not alter the femur mechanical properties of the Δ130–136 strain, which exhibited reduced sensitivity to unloading as evidenced by stiffness (Figure 2H). No significant difference was observed with regard to the elastic modulus (Figure 2I) or maximum stress (Figure 2J) between control and HLU in all three genotypes.

### HLU Increases Cortical Empty Lacunae of Osteocytes in Δ130–136 Mice

**FIGURE 3:**
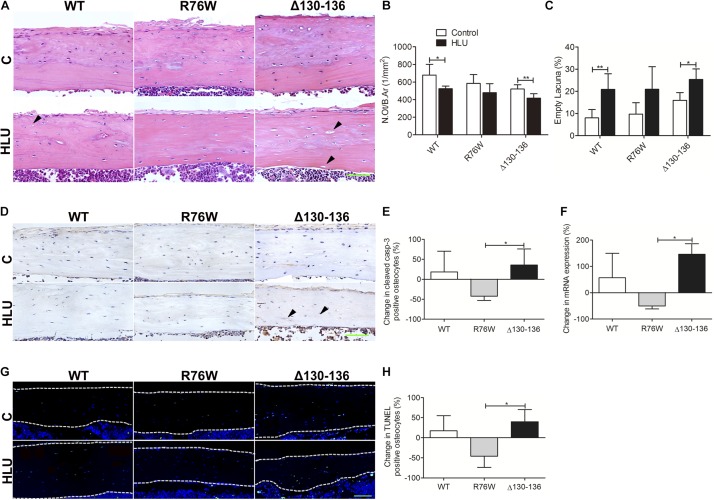
Changes in osteocyte survival in the tibial cortical bone of Cx43 transgenic mice following HLU. **(A)** Representative hematoxylin-eosin-stained tissue sections of tibia cortical bone; solid arrowheads indicate empty lacunae. Osteocyte number (N.Ot/B.Ar) is decreased **(B)** and number of empty lacunae is increased **(C)** in both WT and Δ130–136 mice following HLU. *n* = 5–7/group. Decreased cleaved caspase-3 **(D,E)** and TUNEL signal **(G,H)** are observed in R76W mice but not in the other groups. *n* = 3/group. **(F)** Real-time PCR shows significantly decreased caspase 3 expression in R76W compared with Δ130–136 mice in the cortical bone. White dashed lines indicate bone margins. *n* = 3/group. Scale bar = 60 μm. Comparisons via two-way with Bonferroni test **(B,C)** or one-way ANOVA with Turkey test **(E,F,H)**. Data represent the means ± SD. *P < 0.05, **P < 0.01. HLU, hindlimb unloading; WT, wild-type; R, R76W; Δ, Δ130–136; B.Ar, bone area; BS, bone surface; C, control; N.Ot, osteocyte number; B.Ar, bone area.

Hindlimb unloading decreased N.Ot/B.Ar and increased the number of empty lacunae in WT and Δ130–136 mice, whereas the changes were not significant in R76W mice (Figures 3A–C). Percent change of cleaved caspase 3-positive (Figures 3D,E) and TUNEL-positive osteocytes (Figures 3G,H) was significantly decreased in R76W mice compared to that in Δ130–136 mice during HLU. Real-time PCR also revealed a significant difference in caspase 3 expression between R76W and Δ130–136 mice in cortical bone (Figure 3F).

### Cx43 Hemichannels Affect Periosteal Osteoclastogenesis in Response to HLU

**FIGURE 4:**
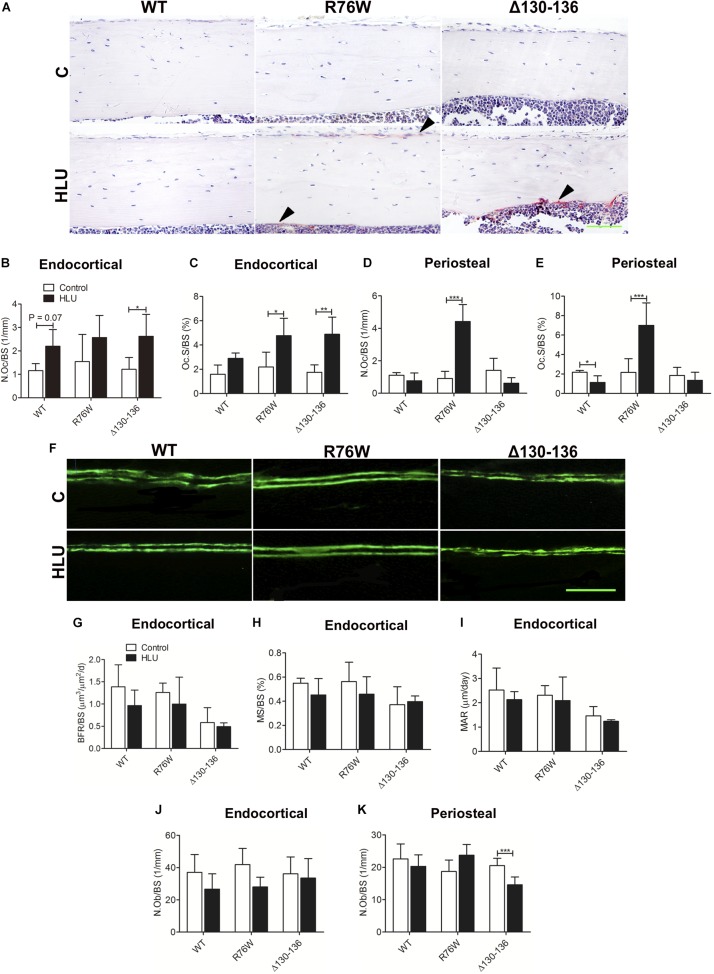
Cx43 affects endocortical osteoclast activity and periosteal bone remodeling in response to HLU. Representative image of tartrate-resistant acid phosphatase staining **(A)** showing increased endocortical osteoclast number and osteoclast area in Δ130–136 mice **(B)** in addition to increased osteoclast area in R76W mice **(C)**. **(D,E)** Periosteal osteoclast activity is not obviously increased in R76W mice. Solid arrowheads indicate tartrate-resistant acid phosphatase-positive osteoclasts. *n* = 3–5/group. **(F)** WT and Cx43 transgenic mice were injected twice with calcein dye. The femur was isolated and plastic sections of the midshaft endocortical bone were prepared. Bone histomorphometric analysis reveals that BFR/BS **(G)**, MAR **(H)**, and MS/BS **(I)** are unchanged during HLU, with unloading having little effect in Δ130–136 mice. *n* = 4–6/group. Scale bar = 200 μm. Endocortical osteoblast number **(J)** is unchanged during HLU; however, periosteal osteoblast number is decreased in Δ130–136 mice **(K)**. *n* = 4–7/group. Scale bar = 60 μm. Comparisons via two-way with Bonferroni test **(B–E,G–K)**. Data represent the means ± SD. *P < 0.05, **P < 0.01, ***P < 0.001. HLU, hindlimb unloading; WT, wild-type; R, R76W; Δ, Δ130–136; N.Oc, osteoclast number; Oc.S, osteoclast surface; BFR, bone formation rate; BS, bone surface; MAR, mineral apposition rate; MS, mineral surface; N.Ob, osteoblast number.

Hindlimb unloading increased tibial endocortical N.Oc/BS and Oc.S/BS in Δ130–136 mice by 116 and 179%, respectively, with a similar increase of Oc.S/BS observed in R76W mice (+118%) (Figures 4A–C). HLU also tended to induce increase Oc.S/BS in WT, although this did not reach significance (Figure 4B, *P* = 0.07). Periosteal N.Oc/BS and Oc.S/BS were obviously increased in R76W but not in WT and Δ130–136 mice (Figures 4D,E).

Calcein double labeling revealed no significant difference between control and HLU groups in all three strains in terms of BFR/BS, MS/BS, and MAR (Figures 4F–I); similar results were obtained by hematoxylin and eosin staining for N.Ob/BS (Figure 4J), except for decreased N.Ob/BS in the periosteal region of Δ130–136 mice (Figure 4K).

### Inhibition of Gap Junctions Affects the Ratio of RANKL/OPG and Sclerostin Expression in Osteocytes Following HLU

**FIGURE 5:**
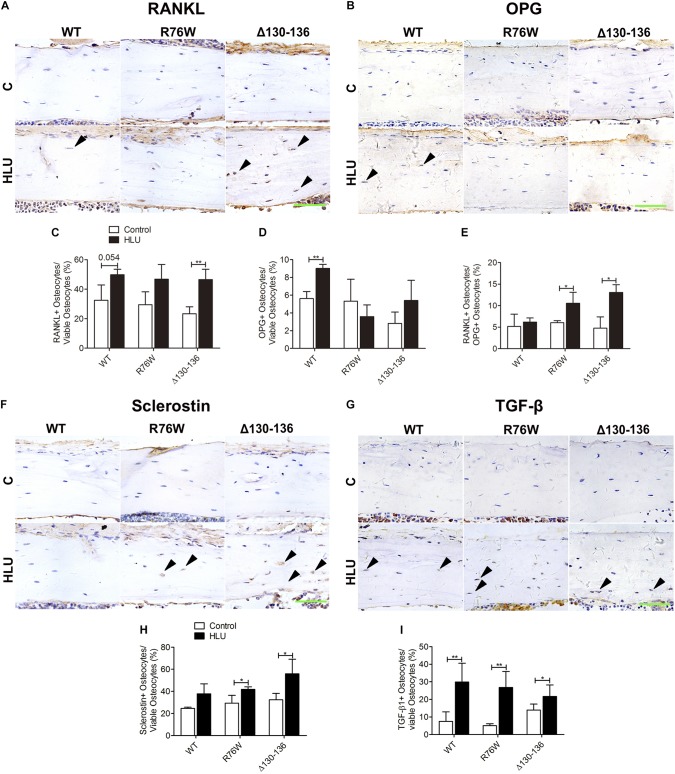
HLU affects cortical osteocyte RANKL/OPG and sclerostin expression in Cx43 transgenic mice. **(A,C)** RANKL-positive osteocyte number is increased in Δ130–136 mice following HLU. **(B,D)** OPG-positive osteocytes number is increased only in WT mice. **(E)** RANKL/OPG-positive osteocyte number is increased in both R76W and Δ130–136 mice. **(F,H)** Sclerostin-positive osteocyte number is increased in the cortical bone of R76W and Δ130–136 mice. **(G,I)** TGF-β1-positive osteocyte number is increased in all three types of mice. Solid arrowheads indicate RANKL-, OPG-, sclerostin-, or TGF-β1-positive osteocytes. *n* = 3/group. Scale bar = 60 μm. Comparisons via two-way with Bonferroni test **(C–E,H,I)**. Data represent the means ± SD. *P < 0.05, **P < 0.01. HLU, hindlimb unloading; WT, wild-type; R, R76W; Δ, Δ130–136.

To clarify the mechanism underlying the alterations in osteoclast and osteoblast activities in the transgenic mice, we detected the expression of bone remodeling markers including OPG, RANKL, sclerostin, and TGF-β1 in osteocytes. In the cortical area, the number of RANKL-positive osteocytes was markedly increased in Δ130–136 mice (98%) tended to increase in WT (*P* = 0.054) by HLU (Figures 5A,C). However, the number of OPG-positive osteocytes was increased only in WT mice (Figures 5B,D). Consequently, the ratio of RANKL-positive/OPG-positive osteocytes was increased only in the mutant strains (Figure 5E). In response to HLU, the expression of sclerostin, a canonical inhibitor of Wnt signaling, was also enhanced only in Cx43-mutant mice (Figures 5F,H). TGF-β1 was enhanced in both WT and transgenic mice (Figures 5G,I).

## Discussion

Hindlimb unloading is a useful model for mimicking bone loss caused by disuse. In the growing skeleton, such cancellous bone architecture is unstable (Iwaniec et al., 2016). In the present study, mechanical unloading via HLU led to deterioration of trabecular bone microstructure both in 10 week old WT and Cx43 mutant mice. However, Tb.Th was significantly altered in the mutants relative to that in WT mice, as evidenced by the larger reduction in SMI (49% in R76W and 28% in Δ130–136 versus 18% in WT), which reflects the number of plates and rods constituting the bone structure. Alternatively, during osteopenia and osteoporosis, trabecular bone changes from plate to rod form, resulting in an increase in SMI. The observation that the two transgenic mouse strains showed more obvious change of SMI and Tb.Th compared with those of WT mice suggested that inhibition of gap junctions but not hemichannels aggravates structural alterations in trabecular bone during unloading.

Conversely, HLU did not cause morphological changes in the cortical bone of WT mice, implying that trabecular bone is more sensitive than cortical bone to unloading, as previously reported in rats (Jia et al., 2014) and mice (Tajima et al., 2018). Clinical studies have also demonstrated that reduced mechanical stimulation causes greater bone loss at trabecular rather than at cortical sites (Tervo et al., 2009). In the present study, we revealed that HLU induced cortical bone loss in transgenic but not in WT mice. This contradicts earlier findings (Lloyd et al., 2012, 2013) of decreased cortical bone mass in both WT and osteoblast-specific Cx43 cKO mice during unloading. We speculated that the difference between WT mice following HLU in our study compared to that of Lloyd et al. (2012, 2013) was due to the age of the animals, as 7 and 10 week old (youth) mice were used in the studies by Tajima et al. (2018) and ourselves, respectively, whereas Lloyd et al. (2012, 2013) used 6 month old (adult) mice, which may cause the different response to unloading in cortical bone. Compared to WT mice, we also found that Cx43 mutants exhibited greater cortical bone loss following HLU, which is not consistent with some previous studies. In particular, OC-Cre cKO mice showed comparable cortical albeit reduced trabecular response to HLU (Lloyd et al., 2012) and Colα1(I)-Cre cKO mice exhibited less sensitivity in cortical bone but similar trabecular response to botulinum toxin A-induced unloading (Grimston et al., 2011). The discrepancy between these models and the Δ130–136 mutants may be due to the difference of temporal and spatial expression of Cx43. OC- and Colα1(I)-Cre cKO mice lack Cx43 in osteoblasts and osteocytes (Grimston et al., 2011; Lloyd et al., 2012) whereas our mutant mouse model supports good Cx43 expression albeit inhibited channel function (Xu et al., 2015). Furthermore, Cx43 performs some channel-independent functions, such as interacting with transcription factors to regulate gene expression and affect cellular growth (Zhou and Jiang, 2014), which may also account for the variable results.

Nevertheless, our transgenic models with overexpression of dominant negative mutants offer a unique opportunity to dissect the specific involvement of the two types of channels by Cx43. Specifically, the 10 kb *Dmp1* promoter has been extensively used to drive expression/deletion primarily in osteocytes (Kao et al., 2013; Kamiya et al., 2016). In previous immunohistochemical studies of Cx43 transgenic mice, we observed mutant Cx43 protein (GFP-labeled) only in osteocytes, not in other bone cell types. However, other studies using the *Dmp1* promoter with fluorescent probes showed expression in osteocytes in addition to other cell types (Kalajzic et al., 2004). This difference may be partially attributed to the different sensitivity of the fluorescent probe used. Some fluorescence signals might be hypersensitive, which may render it difficult to distinguish the difference of expression levels. In addition, *Dmp1* promoter activity may be not specific to a single cell type. However, our previous results showed that our *Dmp1* transgenic model supported overexpression of mutants primarily localized in osteocytes.

In the current study, HLU induced more bone loss in the cortical bone of transgenic but not WT mice. We considered that this occurred because gap junction inhibition results in enhanced osteoclast endocortical bone resorption, enlarged marrow cavity, and decreased Ct.Th and BMD. However, although cortical bone loss was observed in both transgenic strains, only Δ130–136 mice exhibited decreased sensitivity to HLU with respect to mechanical properties. The relationship between bone structure and mechanical properties is non-linear, with relatively small structural change producing a disproportionate decrease in mechanical properties in OC-Cre cKO mice (Lloyd et al., 2012). In our study, the baseline cortical area and thickness were greater in Δ130–136 than those in WT mice (Xu et al., 2015); thus, similar amounts of bone loss caused by HLU may result in less change in mechanical properties. Notably, in WT mice stiffness was significantly decreased although cortical structural bone loss was not observed. We therefore speculated that mechanical properties are not only related to changes in bone structure but also to those in bone components, such as collagen and non-collagen proteins (Wang et al., 2016).

Cleaved caspase 3 functions as the central executor of the apoptotic pathway (Hengartner, 2000). In this study, R76W mice exhibited decreased levels of cleaved caspase 3 along with diminished TUNEL signal in the cortical area, reflective of apoptotic DNA fragmentation (Gavrieli et al., 1992), in contrast to the upregulation of both measures observed in WT and Δ130–136 mice. Moreover, several studies have shown that TGF-β increases apoptosis and the expression of sclerostin in osteocytes (Loots et al., 2012; Notsu et al., 2017). Conversely, mechanical loading on the tibiae of mice can repress TGF-β signaling and further decrease sclerostin expression (Nguyen et al., 2013). In our study, the enhanced numbers of TGF-β1-positive osteocytes were consistent with the increase in sclerostin-positive osteocytes and osteocyte apoptosis in WT and Δ130–136 mice. However, the lack of additional change of TGF-β1-positive osteocytes and empty lacunae in R76W animals further suggested the important role of hemichannels in osteocyte viability during mechanical unloading.

Our previous *in vitro* studies showed that mechanical unloading affects hemichannel opening and prostaglandin (PGE2) release in osteocytes (Jiang and Cherian, 2003; Xu et al., 2017). PGE2 promotes nuclear localization of β-catenin and protects osteocytes from apoptosis (Kitase et al., 2010). Hemichannel impairment blocks the release of PGE2, thereby increasing apoptosis in osteocytes (Plotkin et al., 2001). The enhanced hemichannels in R76W mice may thus protect osteocytes against apoptosis as evidenced by the decrease of caspase 3 expression, TUNEL signal, and osteocyte numbers along with decreased empty lacunae. In comparison, the combined effects of unloading and osteocyte hemichannel impairment likely enhanced osteocyte apoptosis in the cortical bone of Δ130–136 mice by reducing the number of viable osteocytes and increasing the number of empty lacunae.

Except for the decrease of periosteal osteoblast number in Δ130–136 mice, we did not observe additional changes in bone formation or endocortical osteoblast number following HLU, suggesting that the distinct responses of cortical bone in WT and Δ130–136 mice are mainly due to differences in osteoclastogenesis. HLU increased the ratio of RANKL/OPG- and sclerostin-positive osteocytes in transgenic but not in WT mice, which may be related to the increase in endocortical N.Oc and cortical bone loss in the mutants. Both the ratio of RANKL/OPG and sclerostin constitute negatively regulatory markers of bone mass in response to extracellular mechanical stimulation (You et al., 2008; Tu et al., 2012). As the mechanical sensor in bone tissue, osteocytes represent the major source of RANKL (Nakashima et al., 2011) and sclerostin (Poole et al., 2005). In the present study, both R76W and Δ130–136 strains exhibited an increased ratio of RANKL/OPG-positive osteocytes and activated osteoclasts, suggesting that gap junctions in osteocytes regulate unloading-induced bone resorption. Impairment of osteocytic gap junctions inhibits signal transduction between bone cells, resulting in inadequate biological signaling during unloading; this may induce more osteocytes to produce sclerostin or RANKL, thereby stimulating osteoclastogenesis in R76W and Δ130–136 mice. However, periosteal N.Oc was increased by HLU in R76W mice; together with the increased trend of periosteal osteoblast number, this should reflect increased bone remodeling. We considered that these observations are likely related to the ability of enhanced hemichannel function to protect osteocytes against apoptosis in R76W animals.

There are some limitations in the study. First, specific hemichannel inhibitor treatment in R76W mice may be better to further prove the role of hemichannels in osteocytes during mechanical unloading. And the role of PGE_2_ should be further verified in future studies. Second, only 10-week-old mice were used. Based on previous studies, responses of growing and mature bone to loading/unloading are different. Additional studies to investigate the roles of Cx43 channels in adult mice are therefore warranted. Finally, this study only implemented 4 weeks of the HLU protocol whereas HLU of different durations should be evaluated in the future. Such expanded analyses will facilitate a more complete understanding of the role of hemichannels and gap junctions in response to mechanical unloading.

## Conclusion

The results presented here demonstrate that inhibiting osteocytic Cx43 channels promotes the bone loss induced by unloading, mainly in the cortical area. Moreover, hemichannels and gap junctions play distinct roles in this process, with the former protecting osteocytes against apoptosis and promoting periosteal bone remodeling and the latter modulating endocortical osteoclast activity in response to unloading. These findings provide novel insight into the mechanisms underlying unloading-induced bone loss and establish a foundation for developing potential therapeutic strategies for minimizing this detrimental process.

## Data Availability Statement

All datasets generated for this study are included in the article/supplementary material.

## Ethics Statement

The animal study was reviewed and approved by the Northwestern Polytechnical University Institutional Animal Care and Use Committee.

## Author Contributions

DZ, RL, PS, HX, and JJ designed the study. DZ, RL, and GL performed the experiments. DZ, RL, GL, and MC contributed to data collection. DZ, RL, HX, and JJ analyzed the data and interpreted the data. DZ, HX, and JJ drafted the manuscript. HX and JJ critically revised the manuscript and approved the final version of the manuscript. All authors have read and approved the final submitted manuscript.

## Conflict of Interest

The authors declare that the research was conducted in the absence of any commercial or financial relationships that could be construed as a potential conflict of interest.
